# Redesigning Advanced Life Support teaching and assessment using a constructive alignment approach.

**DOI:** 10.12688/mep.20968.1

**Published:** 2025-08-28

**Authors:** Sophie Moll, Nico Tannemann, Margarita Gestmann, Thorsten Brenner, Frank Herbstreit, Cynthia Szalai

**Affiliations:** 1 Department of Anaesthesiology and Intensive Care Medicine, University Hospital Essen, University Duisburg-Essen, Essen, 45147, Germany; 2 Faculty of Medicine, University Duisburg-Essen, Essen, 45147, Germany

**Keywords:** Team OSCE, Advanced Life Support, constructive alignment

## Abstract

Advanced Life Support (ALS) is a crucial component of medical training. Previously, a single-person OSCE-Station (Objective Structured Clinical Exam) was used to assess these skills, focusing primarily on the team leader role and emphasizing theoretical knowledge. However, students demonstrated deficiencies in key algorithm-compliant practical skills, such as cardiac massage, mask ventilation and defibrillator use, and struggled to integrate into a team-based resuscitation approach. To address this, a constructive alignment approach was used to revise the course and offer a guideline-appropriate, three-person resuscitation model. Learning outcomes and assessment targets were aligned with the course activities to increase student engagement and increase desired skill attainment. In the summer semester 2023, students and lecturers were briefed on the new structure of the course and assessment, specific skills were highlighted, and a model video was provided. The OSCE format was adjusted to assess both practical and non-technical skills. In the new setup, each student was randomly assigned one of three roles and assessed using role-specific checklists with defined criteria, focusing on non-technical and practical abilities. Course activity included training and practice in the three-person resuscitation approach. A team OSCE (tOSCE) approach for assessment was used, with one student for each role being examined. Results indicated both subjective and objective markers of satisfaction in course activities and tOSCE results. A team-based OSCE station proves effective for teaching combined practical and non-technical competencies.

## Background and rationale

Advanced resuscitation skills for adult patients are a critical component of the emergency medicine block practicum at our university. These Advanced Life Support (ALS) skills emphasize a team-based approach to managing cardiopulmonary arrest using an evidence-based algorithm ( Michels *et al.*, 2022). All physicians, regardless of specialty or level of training, must be proficient in conducting high-quality resuscitation and either leading or supporting a resuscitation team. Key interventions, including timely chest compressions, timely defibrillation and airway management, are vital to improving patient outcomes. As such, ALS training is an essential aspect of medical training. In our two-week emergency medicine block practicum, students participate in four two-hour sessions focused on teaching ALS skills in alignment with the recommended algorithm ( Michels *et al.*, 2022). Competence was assessed at the end of the 12-week semester through a six minute, single-person resuscitation OSCE (Objective Structured Clinical Examination) station, which serves as a reliable and validated method for evaluating practical skills ( Khan *et al.*, 2013). The OSCE format provides standardized and objective assessment of knowledge and skills across different examiners ( Yang *et al.*, 2022; Zayyan, 2011).

Previously, students were tested primarily in the team leader role at ALS stations, with a greater emphasis on theoretical knowledge and task delegation rather than practical skills. An analysis of OSCE results revealed that students concentrated more on theoretical aspects and less on mastering the practical components of the ALS algorithm. Under the single-person OSCE Station format, tasks were delegated to an accompanying trained assistant, and the candidate's ability to delegate was assessed. From the OSCE station results, while most students demonstrated proficiency in following the algorithm, only just over half performed practical skills—such as effective cardiac massage and defibrillator use satisfactorily. This approach failed to fully assess the students' ability to execute algorithm-compliant manual skills or engage in the team-based approach essential for ALS. Given that all physicians are required to perform high-quality resuscitation, the European Resuscitation Council (ERC) recommends regular training for healthcare providers ( Michels *et al.*, 2022) to maintain expected competencies. To ensure that students meet these rigorous standards, we therefore revised our ALS teaching concept. To develop a new course format, a constructive alignment ( Biggs, 2014; Hays *et al.*, 2015) approach was used. Intended learning outcomes were established based on the recommended skill sets listed by the ERC. Teaching and learning activities both theoretical and practical were designed to promote engagement, understanding and application of the intended learning outcomes. The OSCE station was then redesigned to reflect and adequately assess the intended learning outcomes and reintroduced as a team OSCE (tOSCE).

## Methods

As our intent was to improve the quality of not only teaching ALS principles but to improve assessment of these principles we used a SQUIRE (Standards for Quality Improvement Reporting Excellence in Education) approach to this study. The constructive alignment approach was adapted to within these reporting principles to provide high value teaching and a robust method of assessment of the expected learned competencies. All data were fully anonymized and any information that could potentially allow student identification was excluded, informed consent both verbal and written was obtained for use of the tOSCE results. The study was conducted in accordance with the principles of the Declaration of Helsinki and approved by the local ethics committee of the University Duisburg-Essen: 23-11567-BO.

### Revised course design: Context and intervention

The updated teaching framework for advanced resuscitation training was introduced as part of the Block Practicum Emergency Medicine at the Medical Faculty of the University Duisburg-Essen. In this two-week course, students, in small groups of 10 to 12, were instructed, in four sessions, on both theoretical and practical aspects of advanced life support for adult patients. The curriculum was based on the ERC guidelines, which provide the foundation for modern resuscitation techniques.

After an initial theoretical overview of the ALS algorithm, students were introduced to the necessary equipment and received refresher training on Basic Life Support (BLS) skills. Course content then advanced to algorithm-based chest compressions, effective and safe manual ventilation, and the proper and safe use of a defibrillator. Students practiced these skills in three-person teams, with an emphasis on correct defibrillation technique, airway management, and the administration of drugs through intravenous lines. In addition to reinforcing basic resuscitation skills, dynamic teamwork and non-technical skills—such as effective communication and leadership—were emphasized.

Practical exercises were carried out in teams of three students, each assuming the roles of team leader, team member 1, and team member 2. These exercises took place on an advanced simulation manikin (Ambu Man Advanced Wireless). To further enhance learning, students had access to scripts outlining required learning outcomes for each role, instructional videos (specially developed for the course) adapted for each role, practice quizzes, and additional e-learning resources on resuscitation procedures, international guidelines and equipment management, all made available via the Moodle platform prior to the practicum. This provided students with the opportunity to consolidate their knowledge before the hands-on sessions.

Faculty instructors, who were provided with all course materials and learning objectives through a departmental Teaching App, were required to familiarize themselves with the teaching content to ensure standardized delivery across sessions. Each student practiced all three designated roles throughout the course. Before the final examination, two days were allocated for self-directed learning, supervised by peer tutors, during which students could practice with the manikins, emergency backpacks, and defibrillators.

### Adjustment of OSCE station assessment tasks

Following the course revisions and the updated learning objectives, the OSCE station assessment structure was also modified to better align the intended learning goals with the focus on practical resuscitation measures. Instead of assessing individual performance, the tOSCE station now involved teams of three students, each randomly assigned to one of the following roles: team leader, team ember 1, or team member 2. The six-minute OSCE station evaluated students based on role-specific checklists, including assessments of both technical and non-technical skills such as communication and teamwork. This station was only one of 18 station OSCE exam from various other specialties.

These revised checklists, tailored to each team role, defined clear performance criteria for resuscitation tasks. Team OSCE examiners were provided with electronic feedback tools to assess the quality of key interventions, such as cardiac compressions, defibrillator use, and airway management. The evaluation process was facilitated by handheld devices that scanned students' matriculation numbers and assigned roles, ensuring that the correct student was assessed using the appropriate checklist. To further standardize the process, students wore color-coded high-visibility jackets corresponding to their designated roles, enabling examiners to easily track individual performance. This system ensured that the correct assessment was applied to the correct role, enhancing both the accuracy and fairness of the evaluation process.

### Analysis of the interventions

Systematic data evaluation was performed by analysing the tOSCE results of students during the summer semester of 2023 and winter semester of 23/24 to provide an objective overview of performance and achievement of intended learning outcomes. A breakdown of the results per role (specific assessment tasks and critical skills (CS) was performed. See Table 1 and Table 2. Data was portrayed as the number and proportion of students with the given number of points for each item/skill; shared roles of Team members 1 and 2 are cumulated as the tasks were similar. Critical skills for each position and all roles are also represented. The four critical skills were mainly related to working in a team and recognising and managing the tasks in a resuscitation situation.

**Table 1.** Assessment tasks for each role Summer Semester 23.

Team leader (n = 55)			
Median checklist score (Q1-Q3): 17 (17-18)			
Score	0	1	2
Initial assessment	2 (3.64 %)	37 (67.27 %)	16 (29.09 %)
Informing team members	0 (0 %)	1 (1.82 %)	54 (98.18 %)
Mask ventilation/Oxygen application	1 (1.82 %)	32 (58.18 %)	22 (40.00 %)
Rhythm Analysis	2 (3.64 %)	37 (67.27 %)	11 (20.00 %)
Correct defibrillation	0 (0 %)	7 (12.73 %)	48 (87.27 %)
Application epinephrine	0 (0 %)	11 (20.00 %)	44 (80.00 %)
Airway management	1 (1.82 %)	3 (5.45 %)	51 (92.73 %)
Algorithm appropriate Management	0 (0 %)	8 (14.55 %)	47 (85.45 %)
Team member 1 & 2 (n = 94)			
Score	0	1	2
Rhythm Analysis	4 (4.26 %)	54 (57.45 %)	36 (38.30 %)
Defibrillation	1 (1.06 %)	19 (20.21 %)	74 (78.72 %)
Immediately starting chest compressions	3 (3.19 %)	19 (20.21 %)	52 (55.32 %)
Quality of chest compressions	0 (0 %)	46 (48.94 %)	48 (51.06 %)
No interruption > 30 sec	0 (0 %)	2 (2.13 %)	92 (97.87 %)
Communication with Team leader	0 (0 %)	8 (8.51 %)	86 (91.49 %)

**Table 2.** Student scores for Critical Skills (CS) Summer Semester 23.

Score	0	1
Total (n = 149)		
Clinical decision making	0 (0 %)	149 (100 %)
Situation awareness	0 (0 %)	149 (100 %)
Task management	1 (0.67 %)	148 (99.33 %)
Teamwork	3 (2.01 %)	146 (97.99 %)

The eight assessment tasks were rated using a scoring system where each criterion allowed a maximum of 2 points per task assessed. Clinical tasks were rated on a scale of 0 (not performed), 1 (incomplete), and 2(completed). The four critical skills were rated as 0 (absent) or 1 (present). Examiners were provided with detailed criteria with action words for each task, facilitating ease of assessment. A total of 20 points was possible per checklist. No demographic data was elicited from the checklists as these were not deemed necessary to the evaluation of the new course format.

Student evaluation of each course is performed every semester by the faculty, which provides a subjective overview of the learning and teaching activities of the students themselves. These results were used to compare student responses for the emergency medicine practicum before and after implementation of the revised course: from summer semester 23 (SoSe23) and after the winter semester 23/24 (WiSe23/24). During the winter semester 22/23, some aspects of the learning tasks were changed, but the OSCE format remained as per the previous semester. These results, therefore, do not fully represent the new course and assessment formats. Evaluation criteria were scored using a seven-point Likert scale, from 1: completely unsatisfied to 7: completely satisfied. ( Table 3 and Table 4)

**Table 3.** Assessment tasks for each role Winter Semester 23/24.

Team leader (n = 57)			
Median checklist score (Q1-Q3): 17 (16-18)			
Score	0	1	2
Initial assessment	3 (5,26 %)	17 (29,82 %)	57 (69,91 %)
Informing team members	0 (0 %)	7 (12,28 %)	50 (87,72 %)
Mask ventilation/Oxygen application	4 (7,02 %)	26 (45,61 %)	27 (47,37 %)
Rhythm Analysis	2 (3,51 %)	27 (47,37 %)	28 (49,12 %)
Correct defibrillation	1 (1,75 %)	15 (26,32 %)	41 (71,93 %)
Application epinephrine	2 (3,51 %)	10 (17,54 %)	45 (78,95 %)
Airway management	0 (0 %)	29 (50,88 %)	28 (49,12 %)
Algorithm appropriate Management	2 (3,51 %)	9 (15,79 %)	46 (80,7 %)
Team member 1 & 2 (n = 102)			
Score	0	1	2
Rhythm Analysis	1 (0,98 %)	45 (44,12 %)	56 (54,90 %)
Defibrillation	3 (2,94 %)	38 (37,25 %)	61 (59,8 %)
Immediately starting chest compressions	6 (5,88 %)	32 (31,37 %)	64 (62,75 %)
Quality of chest compressions	8 (7,84 %)	46 /45,1 %)	48 (47,06 %)
No interruption > 30 sec	4 (3,92 %)	15 (14,71 %)	83 (81,37 %)
Communication with Team leader	1 (0,98 %)	21 (20,59 %)	80 (78,43 %)

**Table 4.** Student scores for Critical Skills (CS)) Winter Semester 23/24.

Score	0	1
Total (n = 159)		
Clinical Decision-making	4 (2,52 %)	155 (97,48 %)
Situation Awareness	5 (2,53 %)	154 (97,47 %)
Task Management	2 (0,63 %)	157 (99,37 %)
Teamwork	2 (0,63 %)	157 (99,37 %)

## Results

Results of the objective tOSCE results are illustrated below as well evaluation of subjective student comments on the new course format.

**Figure 1.**
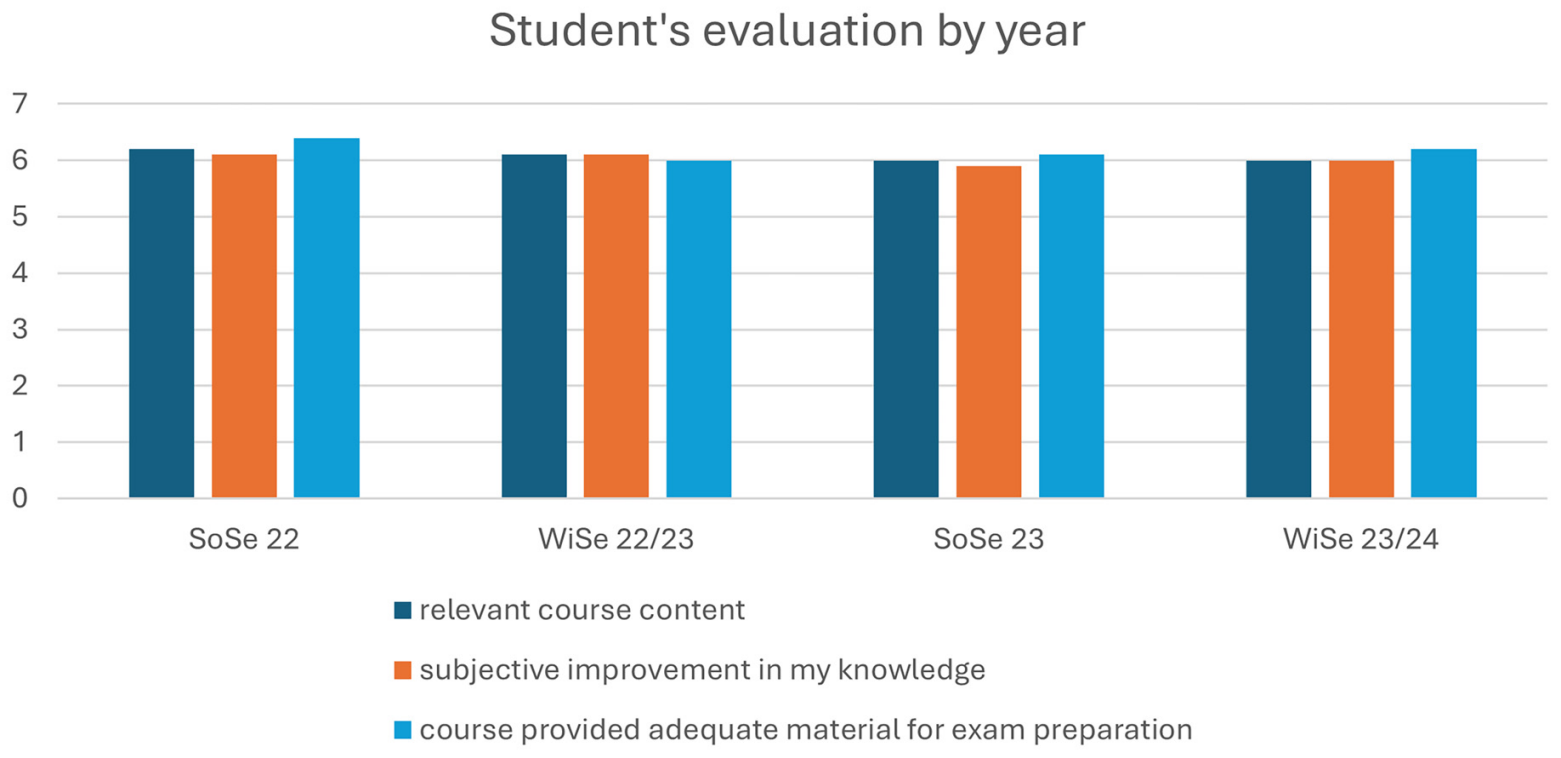
Student evaluation of course content per Semester. SoSe: Summer Semester WiSe: Winter Semester. All figures are property of the authors.

**Figure 2.**
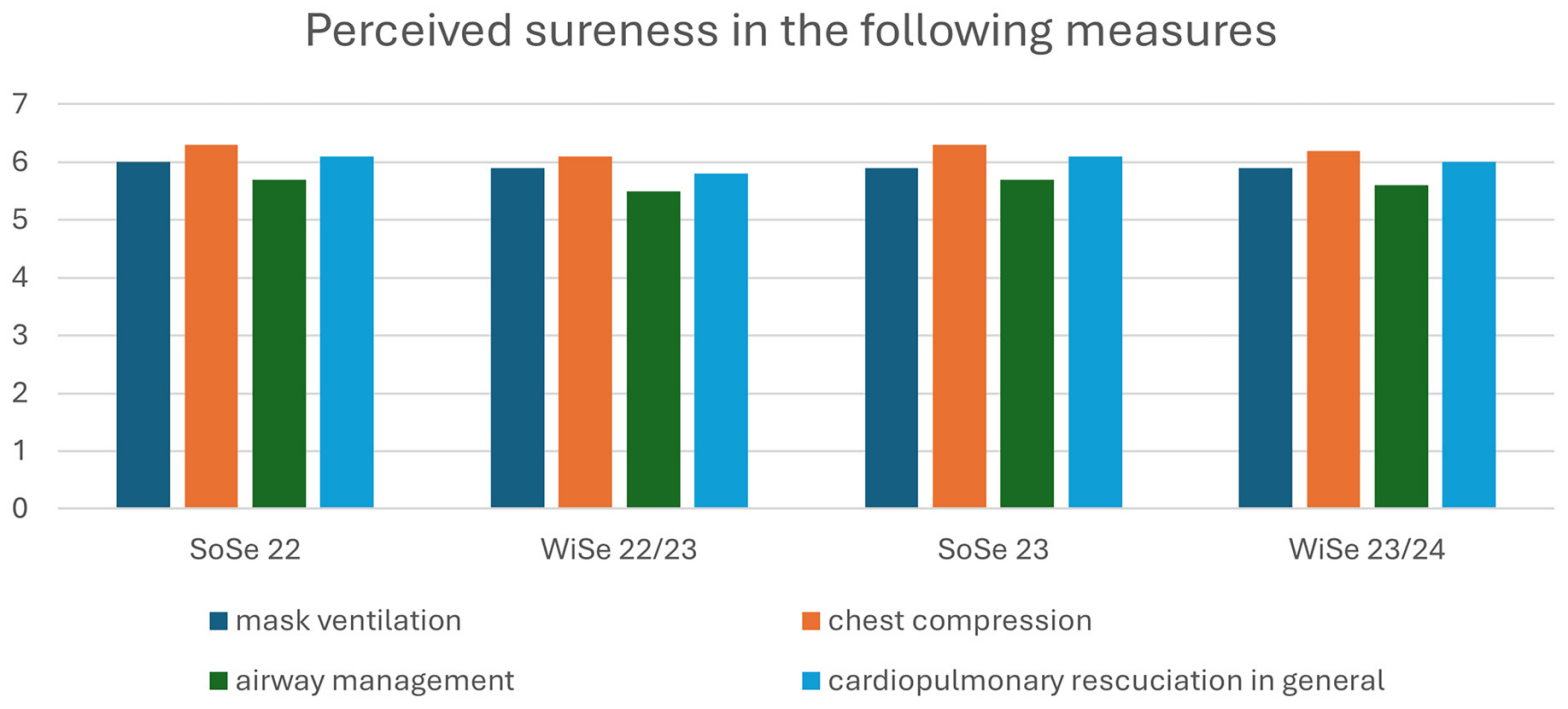
Student self-assessment of skill level of assessment tasks per semester. SoSe: Summer Semester WiSe: Winter Semester. All figures are property of the authors.

Figure 1 shows student ratings of subjective categories over the course of five semesters, before and after the implementation of the new course measures. Figure 2 shows student self-evaluation of their skill levels of the specific intended learning outcomes.

## Discussion

Advanced cardiac life support skills are essential for every healthcare provider. Students and doctors worldwide have indicated that they lack confidence in their competence ( du Plessis *et al.*, 2022; Tadesse *et al.*, 2022) in such skills. It is incumbent on the faculty to teach these life-saving skills effectively and adequately in a setting and format which is as close to reality as possible. Teaching formats and assessment methods should focus on the skills the ERC has identified as essential for effective management. An OSCE format provides a reliable, valid, and objective method to assess the practical and especially the non-technical skills required for effective resuscitation, including targeted communication, knowledge about different task profiles and the ability to function effectively on a resuscitation team. The authors concluded that constructive alignment ( Biggs, 1996; Gilic *et al.*, 2022) was an acceptable approach to align the requisite learning outcomes, teaching activities and assessment tasks. Therefore, we reconsidered the desired outcomes/skills and redesigned the course structure and the OSCE station to reflect these outcomes. As advanced life support is seldom performed by a single person but recommended for a team of at least three persons ( Krzyżanowski *et al.*, 2021), we decided to align the outcomes, activities and assessment tasks to reflect these. Team learning activities and a tOSCE seemed the best tools to reflect these requirements irrespective of the role required.

The authors predicted that as students were required to learn tasks for all three roles, while roles were randomly assigned for the tOSCE, there would likely be reduced student satisfaction and a perceived increased workload with the course. However, the subjective student evaluations were generally positive (almost 6 from a 7-point Likert Scale, similar to previous semesters. Students also rated their perceived skill levels for the four primary learning outcomes: mask ventilation, airway management, chest compressions, and resuscitation. The students reflected no general dissatisfaction with the new course format and assessment.

The tOSCE results showed the ability of the students to perform and communicate in a resuscitation team by low variability and excellent scoring in the performance ratings. The highest scores within the practical skill categories were shown in airway management. The results show that most students demonstrated the desired learning outcomes in skill attainment. However, tasks like chest compressions and rhythm analysis had a higher scoring variability, thus indicating potential for improvement in the learning tasks for these practical skills. These represent areas for consideration when structuring learning/teaching activities for upcoming semesters. It should be noted that although students were satisfied with their skill attainment, practical assessment showed there were areas for improvement. These highlight the importance of aligning student self-assessment, objective assessment tasks and learning outcomes ( Zheng *et al.*, 2024). An informal assessment task during the course may be considered to facilitate accurate student self-assessment. However, the two-week course duration makes the timing of this formative assessment difficult.

Non-technical skills were very well represented in the tOSCE results, emphasising the need for group learning activities and appropriate structured group assessment tasks such as a tOSCE to examine the attainment of these skills ( Schwartzman *et al.*, 2011). Thus, the new course and tOSCE design show that the intended learning outcomes concerning non-technical skills were achieved.

Advanced life support is always administered in a team of at least three persons ( Krzyżanowski *et al.*, 2021); students should be taught using this format. Multi-person bedside teaching assessment ( Deane *et al.*, 2015), as well as team OSCEs (tOSCE), are not novel concepts ( Sharma *et al.*, 2015) but have mainly focused on interprofessional communication rather than practical assessment. Abeyaratne ( Abeyaratne *et al.*, 2024) showed that tOSCE effectively assesses situations requiring shared decision-making and interprofessional education. Although the tOSCE station required more preparation and structuring and was more personal intensive, the benefits of assessing team-based skills should always be considered. Benefits were multiple and included shorter examination days as three students were assigned to one station instead of the usual one. As roles for the OSCE station would be randomly assigned, students reported making more effort to learn a broader range of practical skills and theoretical principles from all three roles than they usually would have for the single-person station. However, no student reported increased stress during the course. Of course, we cannot comment on the transferability of this format to other team-based specialties; however, we can speak to applying the tOSCE to student assessment of other emergency medicine situations as reflected by the promising results.

## Conclusion

Considering all factors, the implementation and promising evaluation of this multi-person tOSCE station show that this format can be used to assess practical and non-technical skills relevant to ALS and is suitable for teaching the skills that make it possible to cope in resuscitation. Further research needs to be conducted to establish the transferability of the tOSCE format to other learning outcomes.

## Ethics and consent

Team OSCE assessment data were collected in a pseudonymized format and subsequently anonymized prior to statistical analysis. Any information that could potentially allow student identification was excluded, informed consent both verbal and written was obtained for use of the tOSCE results. Consent was not possible for the student evaluation as data was collected anonymously, permission to use the data was obtained from the deanery in accordance and the ethics committe. The study was conducted in accordance with the principles of the Declaration of Helsinki and approved by the local ethics committee of the University Duisburg-Essen: 23-11567-BO.

## Glossary of terms

- ALS: Advanced Life Support
- OSCE: Objective Structured Clinical Exam
- tOSCE: Team Objective Structured Clinical Exam
- SQUIRE: Standards for Quality Improvement Reporting Excellence in Education

## Data Availability

**Title:** Redesigning Advanced Life Support teaching and assessment using a constructive alignment approach. **Description:** A blueprint on instructional design and assessment for Advanced Life Support **Contributors:** Cynthia Szalai, Nico Tannemann, Gestmann, Margarita, Frank Herbstreit, and Sophie Moll **Resource type:** Data **Resource language:** English **Funding/Support Information:** None **License:** CC0 1.0 Universal **Affiliated institutions:** Faculty of Medicine, University Duisburg, Essen **Date created:** January 17, 2025 **Date modified:** May 2, 2025 **Subjects:** Medicine and Health Sciences **Repository:** OSF Home: https://osf.io/hzey2/ **Description:** *A blueprint on instructional design and assessment for Advanced Life Support* **License**: *CC0 1.0 Universal* **DOI**: 10.17605/OSF.IO/HZEY2 ( Szalai *et al.*, 2025) **Tags:** Team OSCE, Advanced Life Support, constructive alignment **The project contains the following underlying data:** Report Block Practicum Emergency Medicine SoSe23: 2.7 MB. 2025-04-29 10:14 AM Report Block Practicum Emergency Medicine WiSe2324: 2.6 MB. 2025-04-29 10:14 AM Checklist ALS 103.8 kB 2025-04-29 10:20 AM Results: tOSCE -SoSe23.xlsx 17.7 kB 2025-01-17 03:54 PM Results: tOSCE-WiSe-23-24.xlsx. 28.1 kB 2025-01-17 03:54 PM No clinical data involving human participants was used in this study. All data sets have been de-identified.
