# Supplementary material for: Redesigning Advanced Life Support teaching and assessment using a constructive alignment approach
Source: MedEdPublish (2016). 2025 Aug 28;15:48. [Version 1] doi: 10.12688/mep.20968.1 (PMC12775656; doi:10.12688/mep.20968.1)
Supplement: Peer review history

# Reviewer report: Reviewer response for version 1

**DOI:** 10.21956/mep.22457.r44882

**Published:** 2026-01-06

**Authors:** Shamir O Cawich

**Affiliations:** 1 University of the West Indies, St. Augustine, Trinidad and Tobago

This article addresses a team-based approach to OSCE-Stations to teach ALS content. The authors have shown improvement in the subjective and objective markers of satisfaction in course activities and in OSCE results using this method. The methods appear reproducible. The article reads well, is clearly presented and follows scientific principles.

Have any limitations of the research been acknowledged?

Yes

Is the work clearly and accurately presented and does it cite the current literature?

Yes

If applicable, is the statistical analysis and its interpretation appropriate?

Yes

Are all the source data underlying the results available to ensure full reproducibility?

Yes

Is the study design appropriate and is the work technically sound?

Yes

Are the conclusions drawn adequately supported by the results?

Yes

Are sufficient details of methods and analysis provided to allow replication by others?

Yes

Reviewer Expertise:

HPB Surgery

I confirm that I have read this submission and believe that I have an appropriate level of expertise to confirm that it is of an acceptable scientific standard.

# Reviewer report: Reviewer response for version 1

**DOI:** 10.21956/mep.22457.r44474

**Published:** 2025-11-25

**Authors:** Maria Helena Gaitan

**Affiliations:** 1 University of Quindío, Armenia, Colombia

Excelent work. This perspective can be applied for other topics and scenarios. I appreciate the way it was presented and developed, as well as the likert evaluation, wich revealed the perception of students. The combination of metrics and the perspective of students, reveals a deeper level or learning and teaching stretegies with more effective long-term learning.

Have any limitations of the research been acknowledged?

No

Is the work clearly and accurately presented and does it cite the current literature?

Yes

If applicable, is the statistical analysis and its interpretation appropriate?

Yes

Are all the source data underlying the results available to ensure full reproducibility?

Yes

Is the study design appropriate and is the work technically sound?

Yes

Are the conclusions drawn adequately supported by the results?

Yes

Are sufficient details of methods and analysis provided to allow replication by others?

Yes

Reviewer Expertise:

I am a surgeon, and PhD on Educational Sciences.

I confirm that I have read this submission and believe that I have an appropriate level of expertise to confirm that it is of an acceptable scientific standard.
